# Associations between blood glucose and dynamic plantar pressure distribution in pre-DM patients: a cross-sectional study

**DOI:** 10.3389/fendo.2026.1909052

**Published:** 2026-08-26

**Authors:** Shengbo Wang, Zhoujie Chu, WenXin Li, Jingran Liang, Baitan Cai, Jiaxiang Liu, Zhishan Wang, Wensi Wu

**Affiliations:** 1 School of Traditional Chinese Medicine, Tianjin University of Traditional Chinese Medicine, Tianjin, China; 2 School of Traditional Chinese Medicine, Fujian University of Traditional Chinese Medicine, Fuzhou, China; 3 School of Rehabilitation Medicine, Fujian University of Traditional Chinese Medicine, Fuzhou, China; 4 Department of Preventive Treatment of Disease, The Third Affiliated People’s Hospital of Fujian University of Traditional Chinese Medicine, Fuzhou, China; 5 Emergency Department, The Third Affiliated People's Hospital of Fujian University of Traditional Chinese Medicine, Fuzhou, China

**Keywords:** blood glucose, foot ulcers risk, plantar loading, plantar pressure, Pre-DM

## Abstract

**Background:**

Prediabetes (Pre-DM) confers an elevated risk of peripheral neuropathy and subsequent diabetic foot complications, yet there is a lack of sufficient research on the biomechanical alterations of plantar pressure distribution associated with glycemic abnormalities.

**Objective:**

To investigate the relationship between blood glucose and plantar pressure distributions of 11 zones in Pre-DM patients.

**Methods:**

Fifty-five Pre-DM patients were enrolled. Fasting blood glucose (FBG), glycated hemoglobin (HbA1c), and two-hour postprandial plasma glucose (2hPG) levels were measured. Pressure-time integral (PTI), peak force (PF), and mean plantar pressure (MPP) during walking were recorded using the FreeStep plantar pressure measurement system. Spearman’s rank correlation analysis was used to assess the associations between three glycemic indicators and plantar pressure parameters. A hierarchical linear regression analysis was conducted to examine the association between blood glucose and the related plantar pressure.

**Results:**

Hierarchical linear regression analysis showed that in the first block, BMI was associated with on PF in left HL-L, MPP in left MH2, MPP in left HL-M/L (*p* < 0.01) and MPP in left MF-M (*p*< 0.05). Age was also associated with MPP in left MH2 (*p*< 0.01) and HL-M (*p* < 0.05). In the second block, 2hPG and HbA1c were independently associated with PTI and PF in left MH1, and 2hPG was associated with PF in left HL-L (*p* < 0.05); higher blood glucose levels were associated with increased plantar pressure. In contrast, 2hPG and HbA1c were associated with PF in left T2–5 and in both MH5 (*p* < 0.05); higher blood glucose levels were associated with decreased plantar pressure. FBG had no effect on plantar pressure (*p*>0.05).

**Conclusion:**

Our exploratory study provides the first evidence that 2hPG and HbA1c are independently associated with dynamic plantar pressure in Pre-DM patients. Higher glucose levels are associated with increased loading on left MH1 and heel; conversely, with decreased loading on bilateral MH5 and left T2-5. BMI and age are also the independently associated with dynamic plantar pressure in the left foot.

## 1 Introduction

Diabetes mellitus (DM) is a metabolic disorder characterized by chronic hyperglycemia, which can lead to severe complications including chronic multi-organ damage and neuropathy. By 2050, the global diabetic population is projected to exceed 1.3 billion, with prevalence rates surpassing 10% in most countries, thereby imposing a substantial economic and societal burden (1, 2). Prediabetes (Pre-DM) is an intermediate hyperglycemic state between normal glucose regulation and diabetes, including impaired fasting glucose (IFG), impaired glucose tolerance (IGT), and elevated glycated hemoglobin (HbA1c) levels of 5.7%—6.4% (3). Approximately 5%—10% of Pre-DM patients progress to diabetes annually, while a comparable proportion can achieve reversion to normoglycemia through early intervention (4).

Chronic hyperglycemia is associated with sensory, motor, and autonomic nerves, among which peripheral neuropathy serves as the core cause of foot ulcers and amputation. Several studies have shown that hyperglycemia and oxidative stress directly damage the small vessels that nourish peripheral nerves, leading to ischemia and hypoxia of neural tissue, thereby exacerbating peripheral nerve axonal degeneration and demyelination (5, 6). Furthermore, hyperglycemia can damage both peripheral and central nerves as well as microvessels through multiple metabolic and inflammatory pathways, inducing or amplifying neuropathic pain (7). Peripheral neuropathy induced by hyperglycemia in DM patients can lead to reduced plantar soft tissue thickness, increased tissue stiffness, and an imbalanced forefoot-to-hindfoot load ratio, all of which are directly associated with an elevated risk of ulceration (8, 9). Existing evidence confirms that imbalanced plantar pressure distribution and locally abnormal high pressures are key mechanical factors in the development and recurrence of diabetic foot ulcers (10, 11).

Pre-DM is a critical high-risk stage for the development of type 2 diabetes mellitus (T2DM), during which the complications such as peripheral neuropathy is significantly increased. Plantar pressure assessment, as a non-invasive, quantitative tool for identifying areas of altered loading for diabetic foot (DF), has been widely used for early risk evaluation and precise intervention in diabetic foot during the diabetes stage (12, 13). Given the glycemic changes in Pre-DM and the impact of hyperglycemia on peripheral neuropathy, investigating plantar pressure distribution of individuals with Pre-DM may facilitate earlier warning of DF progression. However, the targeted research remains relatively limited. An early study reported that PF and PTI in the metatarsal region were significantly higher in Pre-DM patients than in healthy controls. However, this study enrolled only 15 Pre-DM subjects, did not perform body weight normalization for plantar pressure parameters, and divided the plantar surface into only five zones without a detailed analysis of statistically related to the metatarsal area (14). Another study applied machine learning algorithms to predict diabetic peripheral neuropathy by combining plantar pressure features (PPP, PPG, PTI) with non-pressure characteristics (age, body weight, height, and HbA1c). But the zone division of plantar pressure in that study is simplified, comprising only three major zones (rearfoot, midfoot, and forefoot), resulting in even less refined zone analysis. Moreover, the study diagnosed Pre-DM solely using HbA1c criteria and thus failed to identify differences in plantar pressure characteristics among different Pre-DM subtypes such as IFG and IGT (15). A recent study indicates that individuals with Pre-DM can also develop DF-related abnormalities such as peripheral arterial disease of the foot, loss of protective sensation (LOPS) symptoms, and foot deformities, which often remain undiagnosed and unmanaged in a timely manner. Furthermore, the study proposes that future research should investigate abnormal plantar pressure distribution patterns in the Pre-DM population (16). Currently, FPG, 2hPG and HbA1c each have their own advantages, disadvantages and limitations in diagnosing Pre-DM. Using a single indicator for the diagnosis of prediabetes can easily lead to missed diagnosis or misclassification, whereas combining all three indicators can significantly improve diagnostic accuracy (17, 18).

Therefore, this study aimed to investigate the correlation between different blood glucose and dynamic plantar pressure of 11 zones in Pre-DM patients, furthermore, preliminarily explore the effects of glycemia on plantar pressure through hierarchical linear regression. We hypothesized that increased blood glucose was related to plantar pressure and three glycemic indicators have different effects on plantar pressure.

## 2 Materials and methods

### 2.1 Participants

Based on a pilot study, using G*Power software (version 3.1.9.7, Heinrich Heine University Düsseldorf, Germany), ρ0;=0.6 (null hypothesis) and ρ1=0.298 (alternative hypothesis), alpha=0.05, power = 0.80 were adopted. The calculation indicated that a total sample size of 55 Pre-DM participants would be required to detect the expected correlation.

A total of 55 Pre-DM patients were recruited who signed informed consent forms. The inclusion criteria were: 1) Adult patients aged 18 years or older; 2) Those meeting the diagnostic criteria for IFG and/or IGT according to the Guidelines for the Prevention and Treatment of Type 2 Diabetes in China (2020 Edition) and voluntarily participating in this study. The Exclusion criteria were: 1) Presence of endocrine diseases other than T2DM, 2) Balance dysfunction caused by medications, intoxication, or other reasons, 3) Presence of joint diseases affecting the hip, knee, or ankle, or limb defects, severe musculoskeletal disorders (such as kyphosis, severe scoliosis, etc.), or gait disorders, 4) Presence of severe psychiatric symptoms such as depression or mania, or severe cognitive impairment, 5) Presence of central nervous system diseases such as Parkinson’s disease, or conditions including vestibular neuritis and acoustic neuroma.

This study was approved by the Ethics Committee of the Third Affiliated Hospital of Fujian University of Traditional Chinese Medicine (Approval Number: 2025KS-58-1).

### 2.2 Measurement of glycemic and plantar pressure

2.2.1 Blood glucose detection: Blood samples were collected from all subjects and sent to the clinical laboratory of the University Affiliated Hospital for biochemical analysis. The main indicators tested included FBG, 2hPG, and HbA1c. Subjects fasted for 8–10 hours prior to blood collection.

2.2.2 Plantar pressure assessment: The plantar pressure were measured using the FreeStep plantar pressure testing system (SENSORMEDICA S.A.S., Italy), which has good reliability and repeatability (19). It consisted of a 1.8 m long pressure testing plate with 1 m extension plates at both ends, a USB module, and analysis software. After becoming familiar with the testing procedure, subjects walked back and forth three times across the pressure platform at a natural gait walked naturally at a speed of 0.97–1.40 m/s without shoes to collect complete dynamic indicators of both feet (20). The average of the three trials was taken. Plantar pressure parameters included the dynamic Pressure-time Integral, Peak Force and Mean Plantar Pressure. All plantar parameters were normalized to body weight (Kg).

### 2.3 Data and statistical analyses

**Figure 1:**
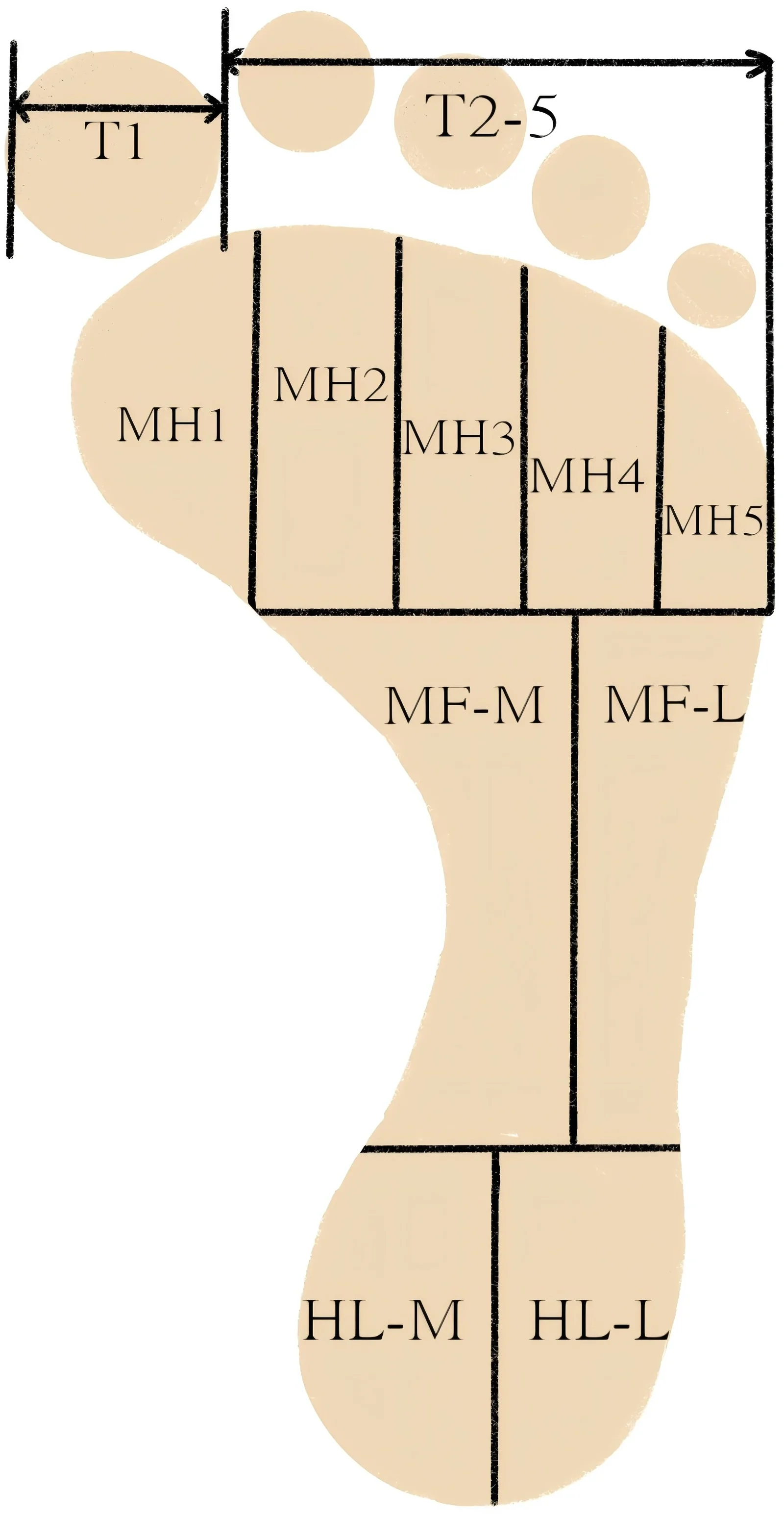
11 zones of plantar pressure.

The plantar foot was divided into 11 zones: T1 (1st toe), T2-5 (2–5 toe), MH1 (1st metatarsal head), MH2 (2nd metatarsal head), MH3 (3rd metatarsal head), MH4 (4th metatarsal head), MH5 (5th metatarsal head), MF-M (midfoot medial), MF-L (midfoot lateral), HL-M (Heel Medial), HL-L (Heel Lateral), as shown in **Figure 1**.

All statistical analyses were performed in SPSS software (SPSS Statistics v27.0, IBM Corp., US).Normality testing was conducted for all continuous variables. Data conforming to a normal distribution were presented as mean ± standard deviation (SD). Data not conforming to a normal distribution were presented as median (P25, P75). The correlations between blood glucose and plantar pressure in each plantar zone were determined using Spearman correlation analysis. To account for multiple comparisons, the Benjamini–Hochberg False Discovery Rate (FDR) procedure was applied to control the false-discovery rate in the correlation analyses. Hierarchical linear regression analysis was performed with the plantar pressure parameters that showed significant correlations as dependent variables and the three glycemic indicators (FBG, 2hPG, HbA1c) as independent variables. Age, sex, and BMI were entered in the first block. In the second block, FBG, 2hPG, and HbA1c were entered separately. Each time a variable was introduced, the change in R² (ΔR²) and its significance for the model were calculated. The unstandardized regression coefficients (B), standardized regression coefficients (β), 95% confidence intervals and P-values for each variable in the final model were reported. A *p*-value<0.05 was considered statistically significant.

## 3 Results

### 3.1 Demographic information

A total of 55 prediabetes’ characteristics are shown in **Table 1**.

**Table 1:** Baseline demographic and clinical characteristics of pre-DM patients.

Index	Value
Gender (M/F)	18/37
Age (y)	54.49 ± 10.19
Height (m)	1.62 ± 0.07
Body weight (kg)	62.04 ± 11.04
BMI (kg/m²)	23.51 ± 3.22
FBG (mmol/L)	6.28 (5.53, 6.62)
2hPG (mmol/L)	8.87 ± 2.74
HbA1c (%)	6.20 (5.80, 6.80)

### 3.2 Relationships between blood glucose and PTI in each plantar zone

In the left foot, FBG (r = 0.274, *p* = 0.043) and 2hPG (r = 0.283, *p* = 0.036) were both positively correlated with PTI in MH1 zone; FBG (r = -0.276, *p* = 0.041) was negatively correlated with PTI in left T1, and 2hPG (r = -0.284, *p* = 0.036) with PTI in MH3.

In the right foot, 2hPG (r = -0.314, *p* = 0.020) was negatively correlated with PTI in MH3.

**Figure 2:**
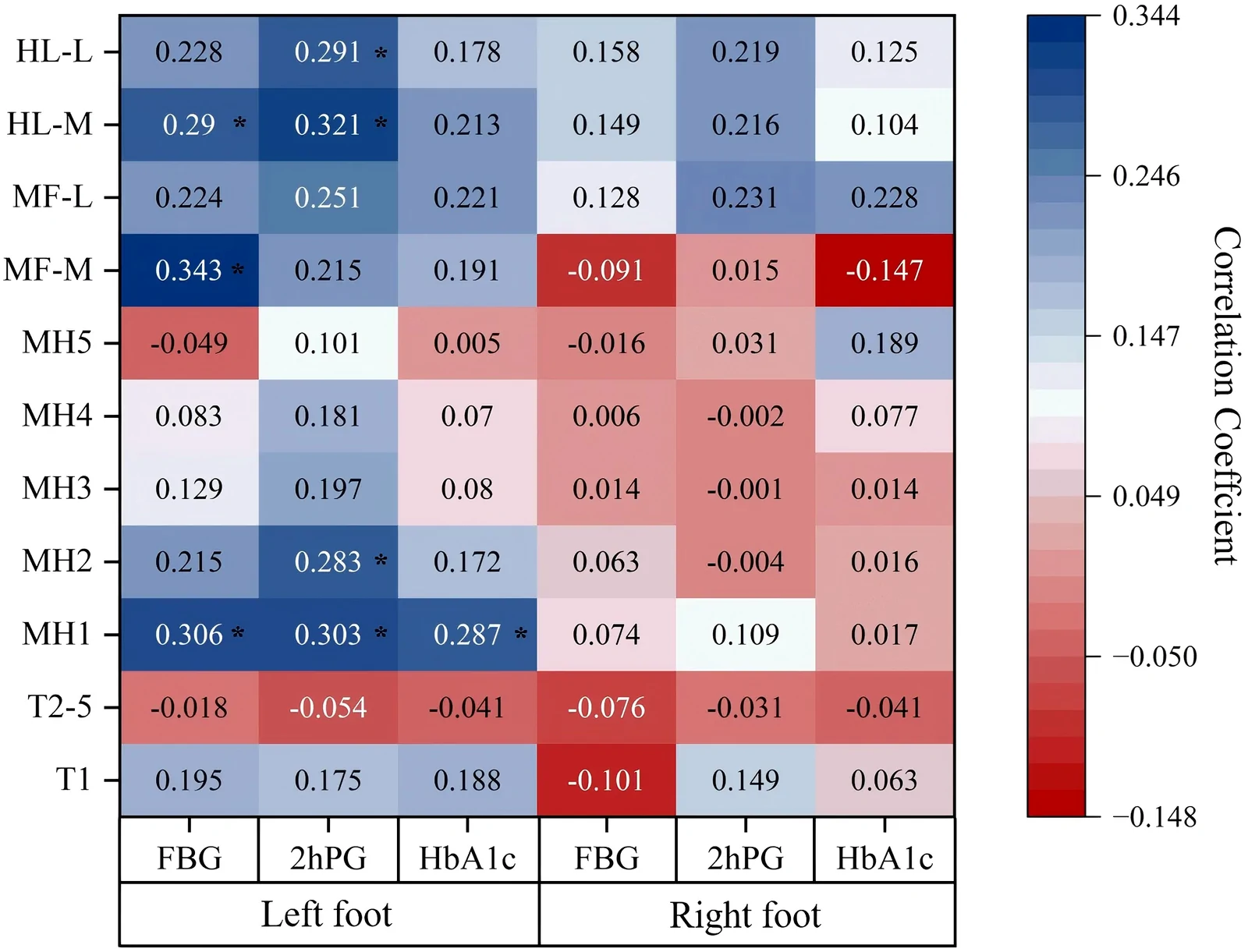
A matrix of the correlations between blood glucose with PTI in each plantar zone. FBG (mmol/L), 2hPG (mmol/L), HbA1c (%), PTI (N·s/BW). *p<0.05, **p<0.01*P<0.01,*P<0.05.

No significant correlations were observed between blood glucose and PTI in the remaining plantar zones (*p* > 0.05) (**Figure 2**).

### 3.3 Relationships between blood glucose and PF in each plantar zone

In the left foot, 2hPG (r = 0.317, *p* = 0.018) and HbA1c (r = 0.282, *p* = 0.037) were positively correlated with PF in MH1, and 2hPG (r = 0.334, *p* = 0.013) with PF in HL-L; FBG (r = -0.276, *p* = 0.042), 2hPG (r = -0.325, *p* = 0.015) and HbA1c (r =-0.463, *p* < 0.001) were negatively correlated with PF in left MH5, and HbA1c (r = -0.355, *p* = 0.008) with PF in T2-5.

In the right foot, 2hPG (r = -0.279, *p* = 0.039) and HbA1c (r = -0.267, *p* = 0.049) were negatively correlated with PF in MH5.

**Figure 3:**
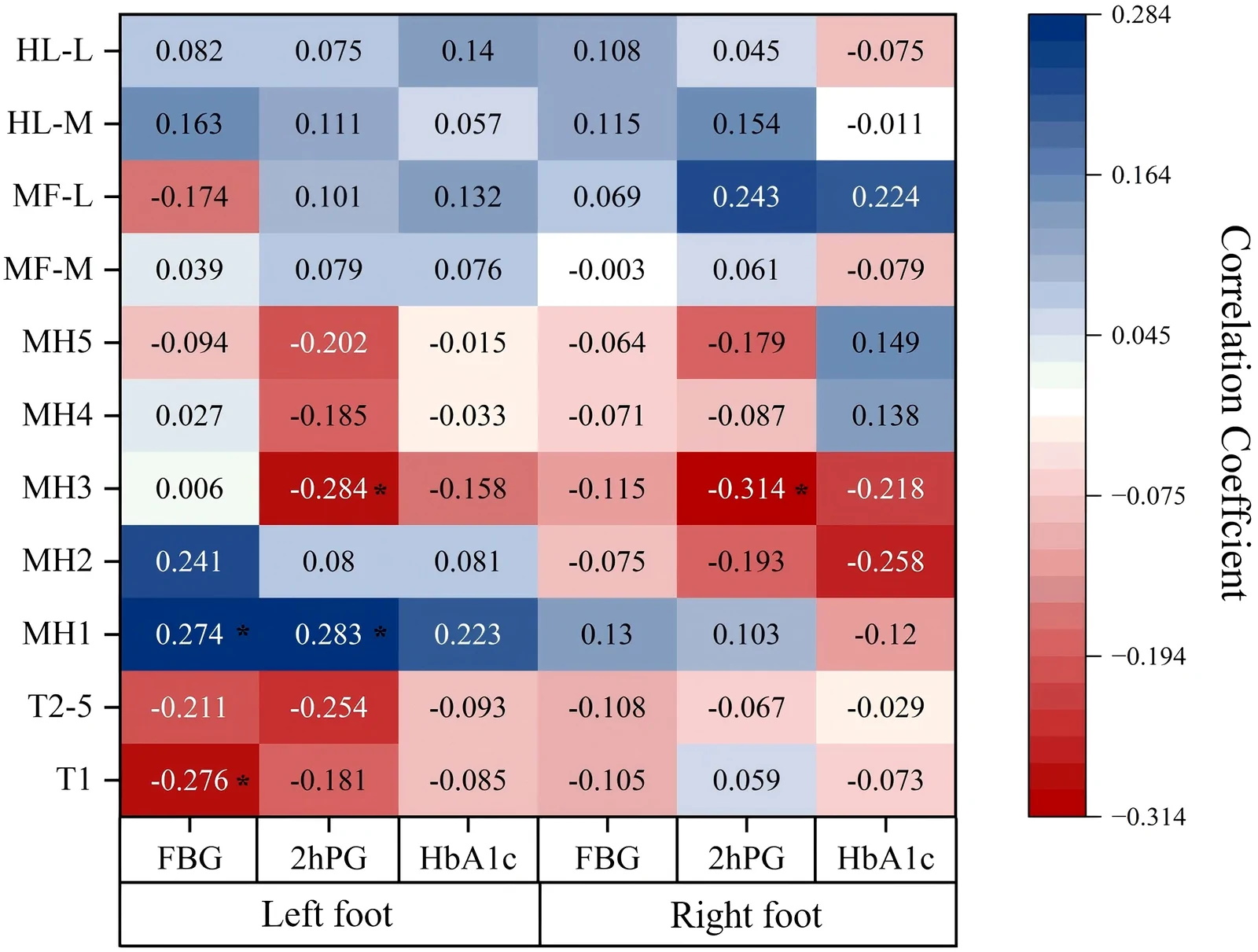
A matrix of the correlations between blood glucose with PF in each plantar zone. FBG (mmol/L), 2hPG (mmol/L), HbA1c (%), PF (N/BW). *p<0.05, **p<0.01 *P<0.01, *P<0.05.

No significant correlations were shown between blood glucose and PF of the remaining plantar zones (*p* > 0.05) (**Figure 3**).

### 3.4 Relationships between blood glucose and MPP in each plantar zone

In the left foot, FBG (r = 0.306, *p* = 0.023), 2hPG (r = 0.303, *p* = 0.025), and HbA1c (r = 0.287, *p* = 0.033) were positively correlated with MPP in MH1; 2hPG (r = 0.283, *p* = 0.036) with MPP in MH2, FBG (r = 0.343, *p* = 0.010) with MPP in MF-M, FBG (r = 0.290, *p* = 0.032) and 2hPG (r = 0.321, *p* = 0.017) with MPP in HL-M, and 2hPG (r = 0.291, *p* = 0.031) with MPP in HL-L.

**Figure 4:**
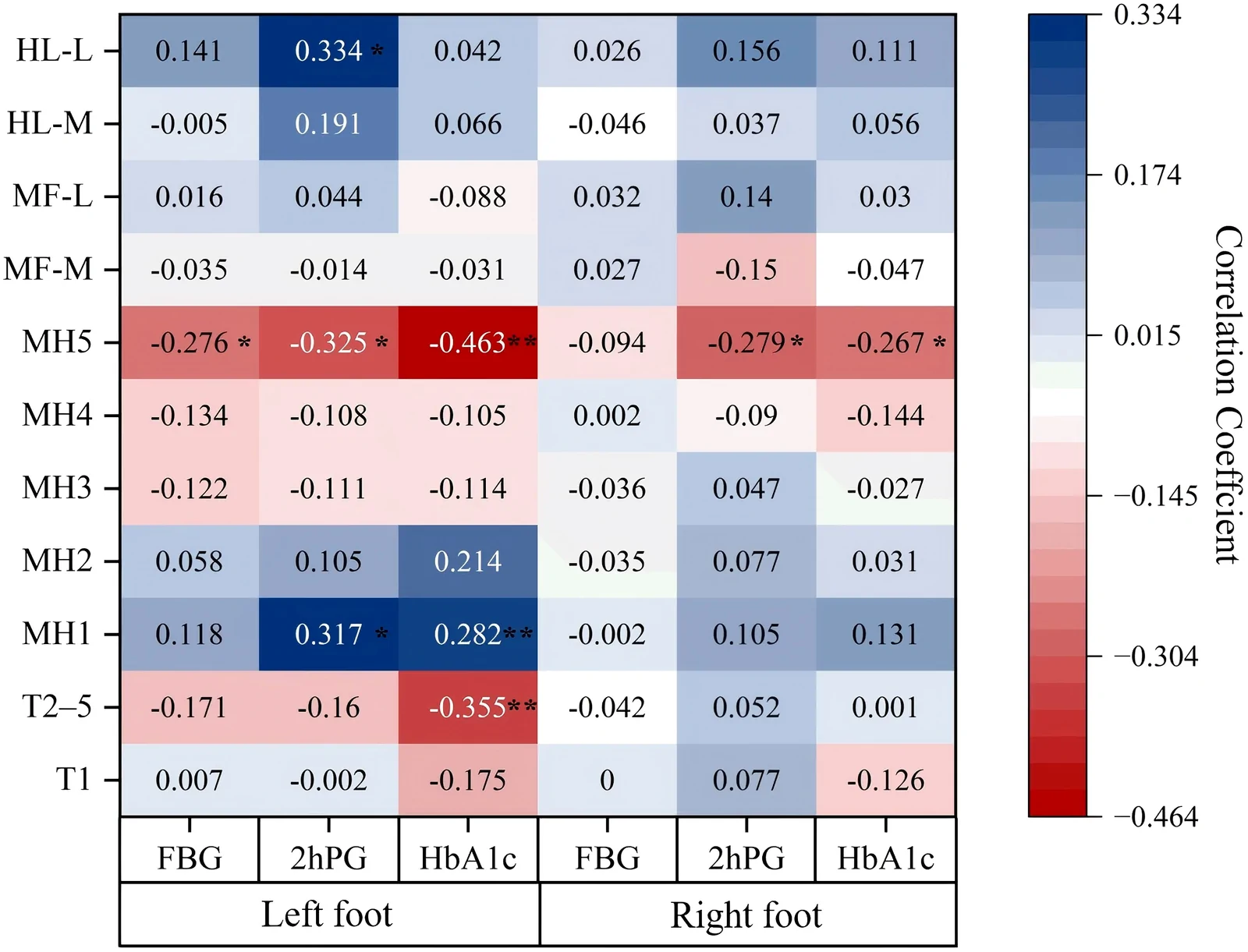
A matrix of the correlations between blood glucose with MPP in each plantar zone. FBG (mmol/L), 2hPG (mmol/L), HbA1c (%), MPP (kpa/BW). *p<0.05, **p<0.01<0.01, *P<0.05.

No significant correlations were shown between blood glucose and MPP of the remaining plantar zones (*p* > 0.05) (**Figure 4**).

### 3.5 Correlation analysis

Spearman correlation analyses were performed to examine the associations between three glycemic measures (FBG, 2hPG, and HbA1c) and three plantar pressure parameters (PTI, PF, and MPP) of 11 plantar zones in both feet. A total of 198 correlation tests were conducted (3 glycemic indicators × 3 pressure parameters × 2 feet × 11 zones). At the nominal significance level (p < 0.05), 22 significant correlations were identified with the three pressure parameters (**Figures 2**–**4**).

To account for multiple comparisons (198 tests), the Benjamini-Hochberg False Discovery Rate (FDR) procedure was applied. After multivariable adjustment and FDR correction (q<0.05), no glycemic indicators remained significantly associated with any of the plantar pressure parameters.

### 3.6 Effects of three glycemic indicators on the plantar pressure parameters showing significant correlations

To exclude confounding factors, hierarchical linear regression analysis was performed. The plantar pressure parameters correlated with blood glucose were sequentially entered as dependent variables, including PTI, PF, and MPP in left MH1; PTI in left T1 and MH3; PTI in right MH3; PF in left T2-5, MH5 and HL; PF in right MH5; MPP in left MH2, MF-M, HL-M and HL-L. In the first block, age, sex, and BMI were entered as covariates. In the second block, FBG, 2hPG and HbA1c were each entered as independent variables. The variance inflation factor (VIF<1.25) for independent variables in all models indicated no serious multicollinearity and the regression results were reliable.

The hierarchical regression analysis showed that in the first block, BMI was independently associated with PF in left HL-L, MPP in left MH2, MPP in left HL-M/L (*p* < 0.01) and MPP in left MF-M (*p* < 0.05). Higher BMI was associated with increased loading in left MF-M and lower loading in left MH2 and HL (*p* < 0.05). Age was also independently associated with MPP in left MH2 (*p*< 0.01) and HL-M (*p* < 0.05). Older age was associated with increased loading in both zones (Appendix 1).

The hierarchical regression analysis showed that in the second block, 2hPG and HbA1c were independently associated with PTI and PF in left MH1, and 2hPG was associated with PF in left HL-L (*p* < 0.05). Higher blood glucose levels are associated with increased loading. In contrast, 2hPG and HbA1c were associated with PF in left T2–5 and in both MH5 (*p* < 0.05). Higher blood glucose levels are associated with decreased loading. FBG had no effect on plantar pressure (*p*>0.05) *(***Table 2***)*.

**Table 2:** A hierarchical linear regression analysis of influencing factors in the second block.

Dependent variable	Independent variable	Model	B	SE	β	*P value*	95% CI
PTI in left MH1	2hPG	Step 2	0.060	0.029	0.290	0.039	0.003, 0.118
PF in left MH1	2hPG	Step 2a	0.110	0.039	0.386	0.007	0.031, 0.188
PF in left MH1	HbA1c	Step 2b	0.320	0.144	0.330	0.031	0.030, 0.610
PF in left HL-L	2hPG	Step 2b	0.113	0.052	0.274	0.034	0.009, 0.217
PF in left T2-5	HbA1c	Step 2b	-0.229	0.088	-0.373	0.012	-0.407, -0.052
PF in left MH5	2hPG	Step 2b	-0.067	0.029	-0.314	0.026	-0.127, -0.008
PF in right MH5	HbA1c	Step 2b	-0.164	0.071	-0.338	0.024	-0.306, -0.022

All results are from hierarchical linear regression analyses adjusted for age, sex, and BMI; total sample size N = 55; β, standardized regression coefficient; SE, standard error; B, unstandardized regression coefficient; 95% CI, 95% confidence interval.

## 4 Discussion

In this study, 11 zones of plantar pressure by body weight−normalized were firstly used to explore the correlation between blood glucose and dynamic plantar pressure distribution in Pre-DM patients. The results revealed that 2hPG and HbA1c were independently associated with increased loading on left MH1 and heel; conversely, with decreased loading on bilateral MH5 and left T2-5. BMI and age are also independently associated with plantar pressure in the left foot.

PTI reflects the cumulative mechanical load during the stance phase of gait and exhibits high sensitivity for the early assessment of DF (21). PF is defined as the maximal vertical ground reaction force during foot-platform contact, which more directly reflects the actual load and provides better stability in samples with large BMI variations (22). MPP represents the force per unit area and comprehensively reflects both the overall load status and changes in contact area (23). Our results showed that FBG, 2hPG and HbA1c were positively correlated with PTI, PF and MPP in left MH1; 2hPG was positively correlated with PF in left HL-L and with MPP in left MH2, HL-M and HL-L; FBG was positively correlated with MPP in left MF-M and HL-M. These findings suggested that as blood glucose increased, the medial loading increased on the left metatarsal head (MH1, MH2) and MF-M, stress especially concentrated on left MH1 and heel in Pre-DM patients. MH1 was a key zone to serves as the primary weight-bearing fulcrum during the propulsive phase of gait, where plantar pressure was abnormally elevated in DM patients (24, 25). Previous studies had confirmed that increased PTI was closely associated with diabetic foot ulcers, and a positive correlation between HbA1c and PF had been observed in high-risk regions such as metatarsal head (26, 27). Another prospective follow-up study showed that as the duration of DM increased, plantar loading gradually shifted from the rearfoot to the forefoot, and increased HbA1c was positively correlated with mechanical parameters in the MH1 zone (26). Combined with our findings, it was speculated that blood glucose association with plantar pressure distribution may be an important mechanism of underlying the high load on MH1 in Pre-DM patients. Abnormal deposition of advanced glycation end products (AGEs) in the plantar fascia and soft tissues led to excessive collagen cross-linking and reduced tissue elasticity (6, 28). on the other hand, subclinical axonal degeneration and demyelination impaired proprioception and muscle control, resulting in abnormally high forefoot loading during propulsion and concentrated stress in the MH1 zone (29, 30). These findings were highly consistent with the classic conclusions of Robinson et al. (14). Microcirculation also played a vital protective role in maintaining homeostasis and tissue defense during injury and inflammation. Notably, microcirculatory impairment had been proposed as a potential missing link in the pathogenesis of foot ulceration in DM patients (31). Recent evidence had suggested that, beyond traditional vascular and metabolic risk factors, endocrine dysregulation serves as a key mediator contributing to vascular dysfunction and tissue vulnerability in DM patients. Furthermore, a constellation of hormonal imbalances compromised vascular integrity and function through multiple pathways, including oxidative stress, inflammatory activation and uncoupling of endothelial nitric oxide synthase (eNOS) (32).

The heel is another key region of high stress and vulnerability to injury in the feet of Pre-DM patients. A Cohort study had shown that during static standing and walking, DM patients exhibited the most significant pressure increases in the heel and certain metatarsal head regions. In addition, both PF and PTI in the heel region are higher than those in healthy young individuals (33). In female DM patients during brisk walking, MPP in the heel region increased significantly with speed, and together with the forefoot, it became a high-pressure area, indicating that heel loading increased markedly during high levels of activity (34). With longer disease duration, DM patients showed a marked increase in PTI in the heel, suggesting that long-term disease duration led to increased cumulative load on the heel (35). Thus, Pre-DM patients may already exhibit an association between glycemic abnormalities and plantar pressure.

Another finding of the study was that in left foot, FBG was negatively correlated with PTI in T1 and PF in MH5, 2hPG with both PTI and PF in MH3, and HbA1c with PF in MH5 and T2-5; In the right foot, 2hPG was negatively correlated with PTI in MH3, both 2hPG and HbA1c with PF in MH5. These findings suggested that elevated blood glucose were associated with decreased plantar loading in lateral metatarsal head (MH3, MH5) of both feet and left toe (T1, T2-5) in Pre-DM patients. The physical stress theory, posited by Mueller et al., suggested that prolonged loading could induce atrophy of the plantar epidermis and soft tissues, thereby impairing their pressure tolerance (36). The concept that had been validated by multiple clinical studies. Previous research showed that in diabetic neuropathy patients without foot ulcers, plantar soft tissue thickness in the MH2–MH4 regions markedly reduced and was negatively correlated with PTI, accompanied by a concurrent decrease in lateral metatarsal loading (37). In DM patients with a history of foot ulcers, progressive plantar tissue atrophy and diminished pressure tolerance rendered the tissue susceptible to injury even at relatively low levels of cumulative tissue stress (38). Several studies had also reported consistent findings that with increasing diabetes duration, overall forefoot (particularly in the medial and central metatarsal regions) pressure or PTI increased, whereas loading in the toe regions decreased or fluctuated (27, 39, 40). That might be attributable to early glycemic abnormalities in DM patients, which could alter peripheral sensation and muscle strength, leading to the gait compensation that shifts loading from the lateral metatarsals and toes to the medial metatarsal (especially MH1). Therefore, our study suggested that plantar loading in Pre-DM patients had already shifted from the lateral MH3–MH5 to the medial metatarsal, transitioning to compensatory loading as blood glucose increased. This also provided an explanation for why foot ulcers at the lateral metatarsal region in DM patients were not associated solely with a single factor of high pressure.

The three sites with the highest incidence of diabetic foot ulcers were the heel, metatarsal heads and T1. Furthermore, the incidence of metatarsal head ulcers was closely associated with higher PF (41). The hierarchical linear regression analysis was performed on all correlated variables. It was found that 2hPG and HbA1c were independently associated with PTI and PF in left MH1, as well as for PF in left T2–5 and bilateral MH5; 2hPG was an independently associated with PF in left HL-L; FBG had no independent effect on plantar pressure. 2hPG is used to assess glucose metabolism and insulin secretion, and to more comprehensively reflect pancreatic β-cell function and insulin sensitivity. More evidence indicated that a sharp postprandial rise in blood glucose induced more intense oxidative stress and endothelial dysfunction, and this intermittent hyperglycemic “spike” might be more direct and significant in damaging neural tissue than FBG (42). 2hPG was more closely associated with inflammatory markers, arterial stiffness, ventricular hypertrophy, and other indices of vascular damage, supporting its central role in the development of complications (43, 44). HbA1c, as a more stable glycemic control indicator, reflects the average blood glucose level over the preceding 2–3 months in Pre-DM patients. Because it is not associated with short-term diet or exercise, it can be used to assess and monitor long-term glycemic control. Previous studies found that HbA1c was associated with an increased risk of diabetic peripheral neuropathy (DPN) and foot complications, and that HbA1c might indirectly influence plantar pressure through neuropathy and foot deformities (45). In patients with cervical spondylosis and diabetes, preoperative HbA1c was an independent risk factor for poor improvement in medial and lateral plantar pressure after two years, with higher HbA1c associated with worse improvement (46).

In addition, the results of this study revealed that the plantar pressure parameters in left foot exhibited a stronger correlation with glycemic indicators, whereas such correlations were less pronounced in right foot. This asymmetry could be attributed to the fact that the left lower limb serves as the dominant weight-bearing side, thereby subjecting the left foot to greater mechanical loading compared to the right foot. Previous studies had reported a significant correlation between soft tissue hardness in MH1 and fasting blood glucose levels in DM patients or Pre-DM patients. Notably, the correlation coefficient was higher for left foot *(*47*)*. A 2-year follow-up revealed that changes in HbA1c, but not fasting glucose, were positively correlated with changes in peak force and peak pressure in left MH1 *(*26*).* Therefore, changes of glycemic indicators may manifest earlier in the plantar pressure parameters of the left foot in Pre-DM patients. Another explanation was that the unilateral pattern may be a common artifact of multiple testing procedures. Further comparative studies on bilateral asymmetry are warranted to validate this finding.

This study has several limitations. First, only 55 patients were enrolled, and healthy individuals as well as patients with type 2 diabetes were not included as controls. In the future, studies with larger sample sizes should be conducted to determine whether the left foot is the main associated side of plantar pressure associated with elevated blood glucose. Second, the correlation analysis entailed a substantial number of comparisons (approximately 200 tests). However, after multivariable adjustment and FDR correction, no blood glucose indicators was independently associated with plantar foot parameters. Accordingly, apart from this single FDR-adjusted significant result, the remaining association-based findings should be regarded as exploratory, pending confirmation in future independent cohorts with larger sample sizes. Third, the sample was predominantly female (37/55), and given the well established sex differences in foot shape and plantar pressure, future research should aim to balance the sex ratio to address this issue. Finally, future research could incorporate multi-scenario assessments involving different movements, speeds, and footwear conditions to enhance the breadth and depth of the investigation. That would provide an evidence-based foundation for the early screening and individualized intervention of diabetic foot ulcers.

## 5 Conclusion

This study found that blood glucose levels were associated with dynamic plantar pressure in Pre-DM patients. 2hPG and HbA1c were the independently associated with increased loading on left MH1 and heel; conversely, with decreased loading on bilateral MH5 and left T2-5. BMI and age were also independently associated with that in the left foot. These exploratory findings generate hypotheses for early identification and intervention strategies for diabetic foot ulcer. Further prospective studies are needed to evaluate clinical relevance.

## Data Availability

The datasets presented in this article are not readily available because the Hospital that approved the study prohibits the authors from making publicly available the research dataset of clinical studies. Requests to access the datasets should be directed to Zhishan Wang sy2024008@fjtcm.edu.cn.
